# Insanity and Allied Neuroses Practical and Clinical

**Published:** 1885-07

**Authors:** 


**Insanity and Allied Neuroses—Practical and Clinical.** By GEORGE H. SAVAGE, M.D., Physician and Superintendent of Bethlem Royal Hospital, Lecturer on Mental Diseases at Guy's Hospital. With nineteen illustrations. Philadelphia: H. C. Lea's Son & Co.

This is one of Lea's clinical manuals or manuals for students, and of the entire series so far published, all have been of more than ordinary value, not only to the student but also for the practitioner. The present volume is a handy-book for study by the student of the subject considered. Dr. Savage's teaching is based largely upon personal experience, which has been extensive, and the physician will find it a book well worthy of perusal.

